# Association between Second-Hand Smoking and Laryngopathy in the General Population of South Korea

**DOI:** 10.1371/journal.pone.0165337

**Published:** 2016-11-18

**Authors:** Haewon Byeon, Dongwoo Lee, Sunghyoun Cho

**Affiliations:** 1 Department of Speech Language Pathology & Audiology, School of Public Health, Nambu University, Gwangju, Republic of Korea; 2 Department of Physical Therapy, School of Public Health, Honam University, Gwangju, Republic of Korea; 3 Department of Physical Therapy, School of Public Health, Nambu University, Gwangju, Republic of Korea; Hokkaido Daigaku, JAPAN

## Abstract

**Purpose:**

The relationship between second-hand smoking and laryngopathy has not yet been reported. Thus, this study investigates the relationship between second-hand smoking and laryngopathy and suggests basic empirical data to prevent laryngopathy.

**Methods:**

This study analyzed 1,905 non-smokers over the age of 19 (269 men and 1,636 women) who completed the health questionnaire, laryngeal endoscope test, and urine cotinine test in the 2008 Korea National Health and Nutrition Examination Survey (KNHANES). Second-hand smoking was defined as a urine cotinine concentration of 50ng/ml and over. Confounding factors included age, gender, education, household income, occupation, alcohol consumption, and coffee consumption. Risk ratios (RR) and 95% confidence intervals (CI) were presented for the relationship between second-hand smoking and laryngopathy by using Poisson regression analysis.

**Results:**

There was a significant relationship between second-hand smoking and laryngopathy (p<0.05). After all compounding factors were adjusted, non-smokers exposed to second-hand smoking had a 2.5 times (RR = 2.47, 95% CI: 1.19–5.08) significantly higher risk of laryngopathy than non-smokers not exposed to second-hand smoking (p<0.05).

**Conclusion:**

In this epidemiological study, there was a significant relationship between second-hand smoking and laryngopathy. More effective anti-smoking policies are required to protect the health of both non-smokers and smokers.

## Introduction

Smoking is likely to have adverse effects on the health of both smokers and non-smokers. Second-hand smoking is defined as the involuntary inhalation of smoke from smokers’ cigarettes [1]. Worldwide, one out of every three non-smokers is known to be exposed to second-hand smoking [2], and in Korea, a 2010 national survey reported that 39.7% of the adult population is exposed to second-hand smoking [3]. According to the U.S. Department of Health and Human Services [4], exposure to second-hand smoking is related to damage of the respiratory system and pulmonary function, such as lung cancer and asthma. Moreover, it was reported that the number of lung cancer deaths from second-hand smoking from 1965 to 2014 was as high as 263,000, while production loss from second-hand smoking exceeded US$56 billion as of 2006 [4].

Second-hand smoking is highly likely to have adverse effects on the larynx located in the upper airway as well as the respiratory system. Nevertheless, studies on the harm of second-hand smoking have mainly focused on its relationship with cancer or respiratory diseases [5–7], while there have been few epidemiological studies on voice problems based on the general population [8, 9]. Moreover, the relationship between second-hand smoking and laryngopathy has not yet been elucidated. As laryngopathy have a high possibility of recurrence even after successful operation [10], the investigation and prevention of risk factors is important, and this requires prior quantitative research on risk factors for populations in local communities.

Research on exposure to second-hand smoking is classified into evaluations by self-report questionnaires and biological evaluations. Even though self-report questionnaires can be used to assess second-hand smoking without measuring tools in a simple manner, they have low reliability due to false responses and recall bias. On the other hand, the urine cotinine test mainly used for biological evaluations is effective in measuring second-hand smoking, as it has a half-life of 18–20 hours and it is a non-invasive method, making it simple to collect samples [1].

Although preceding studies have reported the relationship between smoking and laryngopathy [11], no epidemiological study on the relationship between second-hand smoking and laryngopathy has yet been reported. In addition, studies on exposure to second-hand smoking have been mainly limited to questionnaire surveys on patients [5, 12], and studies that have investigated the exposure of the general population to second-hand smoking with biological tests are rare.

This study investigates the relationship between second-hand smoking and laryngopathy by using the urine cotinine test, and it provides basic empirical data to prevent laryngopathy.

## Methods

### Data Source & Participants

This is a cross-sectional study using a secondary data analysis. The participants of this study were adults, 19 years and older, who participated in the 2008 Korea National Health and Nutrition Examination Survey (KNHANES, a nationwide representative survey of the non-institutionalized population in the Republic of Korea) and who then participated in a health interview, urinary cotinine test, and laryngoscope examination [13]. The survey conformed to the principles outlined in the Declaration of Helsinki and received clearance from the Institutional Review Board (No. 2010-02CON-21-C) of the Korean Center for Disease Control and Prevention (K-CDC). Participants were given identification numbers and guaranteed anonymity. After the survey had been fully explained and all participants had provided written informed consent. The KNHANES is a nationwide cross-sectional survey conducted annually by the K-CDC using a rolling sampling design that involves a complex, stratified multistage probability cluster survey of a representative sample of non-institutionalized civilians in the Republic of Korea. The sampling methods of the KNHANES are described in detail elsewhere [13]. Briefly, the KNHANES 2008 was conducted on 12,528 people from 4,600 households, and the participation rate was 80.8% (n = 9,744).

**Fig 1:**
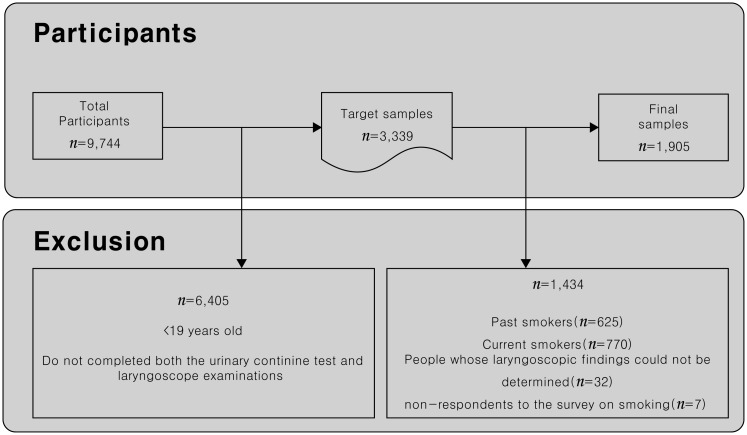
Inclusion and exclusion flow chart of study.

This study targeted 3,339 people who completed both the urinary cotinine test and laryngoscope examinations. Among them, 1,395 smokers (625 past smokers, 770 current smokers), 32 people whose laryngoscopic findings could not be determined, and seven non-respondents to the survey on smoking were excluded from the research, and 1,905 non-smokers (269 men, 1,636 women) were analyzed (Fig 1).

### Measurement

#### Laryngoscope examination

Trained medical staff performed the laryngoscope examinations at a mobile examination center. Endoscopic laryngeal examinations for laryngeal pathologies were performed by an otolaryngologist with a 70° endoscope on female adults. The laryngeal examinations were done with the collaboration of the Korean Society of Otorhinolaryngology-Head and Neck Surgery, which provided technical advice and highly trained otolaryngologists. Based on the data from the laryngoscopic examinations, laryngopathy included the following: vocal nodules, vocal polyps, intracordal cysts, Reinke’s edema, laryngeal granuloma, sulcus vocalis, laryngeal keratosis, laryngitis, laryngeal papilloma, and suspected malignant neoplasm of the larynx.

#### Urine cotinine test

Study subjects’ 5–20ml urine samples were collected, and the cotinine concentration of the collected urine samples was measured using a mass selective detector (GC-MSD, Clarus 600T; Perkin Elmer, Waltham, MA, USA). Standards for defining second-hand smokers have been presented by preceding studies in diverse ways, from 50–100ng/ml [14, 15]. Since the cut-off value for cotinine concentration to judge exposure to second-hand smoking must consider the ethnicity and smoking rate of the related country [16], this study defined 50ng/ml as a cut-off value to judge exposure to second-hand smoking by referring to preceding studies on urine cotinine tests of Korean citizens [17, 18].

#### Confounding factors

Age, education level, income, occupation, alcohol consumption, and coffee consumption were examined. Age was classified as 19 to 29 years, 30 to 39 years, 40 to 49 years, 50 to 59 years, and over 60 years old. Education levels were classified as below elementary school graduation, middle school graduation, high school graduation, and above college graduation. Levels of income for households were classified into four quartiles. Occupations were reclassified into economically inactive population (unemployed person, homemaker), non-manual worker (managers & professionals, clerical support workers, service & sales workers), and manual worker (skilled agricultural & forestry & fishery workers, craft & plant and machine operators and assemblers, and unskilled laborers) occupations. Referring to the standard of the International Center for Alcohol Policies (2005) [19], binge drinking was defined as alcohol intake of 61g for men and 42g for women per drinking session. Coffee consumption was classified into less than one cup a day and more than two cups a day.

### Statistical Analysis

Subjects’ general characteristics, second-hand smoking prevalence, and prevalence of laryngopathy were presented in percentages. The subjects’ general characteristics based on laryngopathy and difference of average urine cotinine concentration were verified by a chi-square test and independent t-test, respectively. Poisson regression was used to assess the relationship between second-hand smoking and laryngopathy, and in order to investigate the independent effect of second-hand smoking on laryngopathy, risk ratios (RR) and 95% confidence intervals (CI) were suggested after adjusting covariates. Sociodemographic variables (age, education, household income, occupation) were adjusted for Model 1, and all confounding variables, including coffee consumption, were adjusted for Model 2. IBM SPSS version 23.0 (IBM Inc., Chicago, Illinois) was used for analyses, and the significance level was 0.05 for the two-sided test.

## Results

### General Characteristics of Subjects

The general characteristics of subjects are presented in Table 1. The average age was 49.8 (standard deviation: 16.4), and there were more females (85.9%) than males (14.1%). Large proportions of the subjects were primary school graduates and lower (35.9%), were non-economically active (47.7%), did not practice binge drinking (87.1%) and drank one cup of coffee or less per day (71.1%). Of the subjects, 6.8% turned out to be second-hand smokers with urine cotinine concentrations of 50ng/ml and over. The prevalence of laryngeal disease was 5.1%.

**Table 1:** The general characteristics of subjects (*n* = 1,905).

Characteristics	*n* (%)
Age, Mean±SD	49.8±16.4
Gender	
Male	269 (14.1)
Female	1,636 (85.9)
Income	
1^st^quartile	425 (22.8)
2^nd^quartile	485 (26.1)
3^rd^quartile	495 (26.6)
4^th^quartile	455 (24.5)
Education level	
Below elementary school graduation	684 (35.9)
Middle school graduation	202 (10.6)
High school graduation	612 (32.2)
Above college graduation	405 (21.3)
Occupation	
Economically inactive population	907 (47.7)
Non-manual worker	481 (25.3)
Manual worker	513 (27.0)
Problem drinking	
No	1,660 (87.1)
Yes	245 (12.9)
Coffee consumption	
One cup and less a day	1,211 (71.1)
More than 2 cups a day	492 (28.9)
Urine conitine test	
<50ml	1,775 (93.2)
≥50ml	130 (6.8)
Laryngopathy	
No	1,807 (94.9)
Yes	98 (5.1)

### Characteristics of Subjects based on Laryngopathy

Subjects’ general characteristics based on laryngopathy are presented in Table 2. According to the results of the chi-square test, there were no significant differences based on laryngopathy in any of the variables.

**Table 2:** Subjects’ general characteristics based on laryngopathy, n (%).

Characteristics	Laryngeal disease (n = 1,905)		p
	No (n = 1,807)	Yes (n = 98)	
Age			0.188
19–29	236 (95.5)	11 (4.5)	
30–39	324 (95.6)	15 (4.4)	
40–49	340 (96.3)	13 (3.7)	
50–59	335 (92.5)	27 (7.5)	
60+	572 (94.7)	32 (5.3)	
Gender			0.066
Male	249 (92.6)	20 (7.4)	
Female	1558 (95.2)	78 (4.8)	
Income (quartiles)			0.637
Q1	399 (93.9)	26 (6.1)	
Q2	459 (94.6)	26 (5.4)	
Q3	474 (95.8)	21 (4.2)	
Q4	432 (94.9)	23 (5.1)	
Occupation			0.709
Economically inactive population	861 (94.9)	46 (5.1)	
Non-manual worker	453 (94.2)	28 (5.8)	
Manual worker	489 (95.3)	24 (4.7)	
Education levels			0.085
≤ Elementary school graduation	641 (93.7)	43 (6.3)	
Middle school graduation	198 (98.0)	4 (2.0)	
High school graduation	584 (95.4)	28 (4.6)	
≥ College graduation	382 (94.3)	23 (5.7)	
Binge drinking			0.420
No	1,572 (94.7)	88 (5.3)	
Yes	235 (95.9)	10 (4.1)	
Coffee consumption			0.357
Less than one cup a day	1,149 (94.9)	62 (5.1)	
More than two cups a day	472 (95.9)	20 (4.1)	
Second-hand smoking			0.173
No	1,687 (95.0)	88 (5.0)	
Yes	120 (92.3)	10 (7.7)	

### Relationship between Second-Hand Smoking and Laryngopathy

The relationship between second-hand smoking and laryngopathy is presented in Table 3. According to the results of the Poisson regression, second-hand smoking had a significant relationship with laryngopathy (p<0.05). After all confounding variables were adjusted (Model 2), non-smokers exposed to second-hand smoking had a 2.5 times significantly greater risk of laryngopathy than non-smokers not exposed to second-hand smoking (RR = 2.47, 95% CI: 1.19–5.08, p<0.05).

**Table 3:** The relationship between second-hand smoking and laryngopathy: Poisson regression, risks ratio and 95% CI.

Variables	Crude model	Model 1	Model 2
NES	1	1	1
NNES	1.59 (0.81, 3.15)	1.79 (0.89, 3.59)	2.47 (1.19, 5.08)*

*p<0.05

NES = Non-smokers exposed to second-hand smoking; NNES = non-smokers not exposed to second-hand smoking. Model 1: adjusted for age, gender, education, household income, and occupation. Model 2: additionally adjusted binge drinking and coffee consumption.

## Discussion

Since laryngopathy have a high possibility of recurrence, it is necessary to clarify and prevent their risk factors. In the investigation on the relationship between second-hand smoking and laryngopathy in this study, even after confounding variables were adjusted, non-smokers exposed to second-hand smoking had a 2.5 times significantly greater risk of laryngopathy than non-smokers not exposed to second-hand smoking. Although it is difficult to compare the results of this study directly, numerous studies on non-smokers living with smokers have reported that second-hand smoking not only is related with lung cancer and laryngeal cancer but also increases the risk of respiratory organ infection [12, 20].

According to a longitudinal cohort study that traced 97,972 patients in Denmark, Finland, Iceland, Norway, and Sweden for 45 years from 1961 to 2005 [5], waiters (who have a high possibility of occupational exposure to tobacco smoke) had a 2–5 times significantly greater risk of laryngeal cancer and a 2–4 times significantly greater risk of oral cancer than the general population. A population-based case-control study also reported a similar result: Non-smokers exposed to second-hand smoking had a 1.2 times greater risk of laryngeal cancer [7] than those who were not.

Another study also reported a correlation between second-hand smoking and respiratory diseases. According to this cohort study that traced 3,914 adults over the age of 25 for 10 years, those who were exposed to second-hand smoking had a 1.7 times greater risk of airway obstructive diseases [21]. In addition, the risk of death from emphysema or chronic bronchitis for non-smoking women who lived with smoking husbands was 29% higher than that for women who lived with non-smoking husbands [20]. There was also a report that men and women exposed to second-hand smoking had a 1.2 and 1.1 times greater risk of chronic cough, respectively [22].

A large-scale epidemiological study by Jee et al. [12] implied a dose–response relationship of second-hand smoking, and in a cohort study on 160,130 Korean women aged 40–87, women living with currently smoking husbands had a significantly higher risk of lung cancer from second-hand smoking than women living with non-smoking husbands. Moreover, when the husbands had smoked for 30 years or more, women’s risk for lung cancer was as much as 3.1 times higher.

Under this background, after comprehensively analyzing preceding studies on second-hand smoking, the Environmental Protection Agency of the U.S. set second-hand smoke as a “Class A” carcinogen together with carcinogenic materials such as arsenic, asbestos, and radioactive materials [23]. In addition, the 2006 U.S. Surgeon General’s report reemphasized the relationship between non-smokers’ exposure to second-hand smoking and lung cancer [24], and a recent meta-analysis that investigated the relationship between second-hand smoking and lung cancer in 87 epidemiological studies [25] also confirmed the significant relationship between second-hand smoking and lung cancer.

The relationship between second-hand smoking and laryngopathy is deemed to be caused by toxic materials in side-stream smoke, which have adverse effects on the larynx. Cigarette smoke is classified into main-stream smoke, which is emitted by the smoker after inhaling the smoke, and side-stream smoke, which comes out of the incinerated cigarette itself, and 80% of the smoke inhaled by second-hand smoking is side-stream smoke [26]. According to the National Toxicology Program, at least 250 chemicals contained in second-hand smoke are toxic materials that are more concentrated in side-stream than in main-stream smoke, and among them, concentrations of carcinogens, such as benzene, 2-naphthylamine, and benzopyrene, are at least 2 times and up to 30 times higher in side-stream than in main-stream smoke [27]. Benzene and benzopyrene are known to cause inflammatory change by stimulating the mucous membrane of the vocal cords, and in the report of the National Cancer Center of Korea that analyzed the concentration of main-stream smoke and side-stream smoke, side-stream smoke had 30 times more benzene, 50 times more formaldehyde, 3.5 times more benzopyrene, and 7.2 times more cadmium than main-stream smoke [28].

The causal relationship in which smoking has a direct effect on the change of the mucous membrane of the vocal cords has been verified by numerous animal experiments [29]. Rats exposed to second-hand smoking had significantly higher proportions of moderate and focal inflammation of the larynx than a control group [30]. Some animal experiments have confirmed the harm of side-stream smoke: Rats exposed to side-stream smoke had a 6 times greater risk of cancer than those exposed to main-stream smoke [31].

When the toxic materials included in second-hand smoke are inhaled by non-smokers, they are highly likely to have adverse effects on the respiratory system. Although there has been no report so far on the effect of second-hand smoking on the upper respiratory tract and larynx, it is likely that second-hand smoking has adverse effects on laryngopathy considering that the mucous membranes of vocal cords are highly likely to be exposed to the toxic materials contained in side-stream smoke. The results of this epidemiological study support the relationship between second-hand smoking and laryngopathy. Prospective cohort studies are required in the future to investigate the causal relationship between second-hand smoking and laryngopathy. This study’s strength lies in the fact that it is an epidemiological study that verified the relationship between second-hand smoking and laryngopathy for the first time by using diagnostic data representing the population of local communities. However, limitations of this study are as follows. First, due to inter-individual variability for cotinine, even when they were exposed to the same level of second-hand smoking, those with less than 50ml of urine cotinine concentration might have been classified as non-smokers not exposed to second-hand smoking. Second, since urine cotinine has a short half-life, even among subjects frequently exposed to second-hand smoking, such subjects might have been classified as non-smokers in the urine cotinine test if three days had already passed since their last exposure to second-hand smoking at the time of the test. Third, although this study adjusted various covariates, such as sociodemographic variables and health behaviors, it did not control potential confounding factors, such as laryngopharyngeal reflux [32], voice abuse and misuse [33], and noise environment [34], which have been reported as relevant to laryngopathy in the preceding studies. Fourth, this study showed that the risk ratio associated with the relevance of passive smoking and laryngopathy was higher in the adjusted model than in the crude model. Moreover, it was only significant in the adjusted model. We could not exclude the possibility that the significant relationship between passive smoking and laryngopathy was due to the confounding effect. Fifth, it is not possible to determine the exposure period of second-hand smoking with urine cotinine concentration alone. An investigation of the dose–response relationship between second-hand smoking and laryngopathy is required in the future. Sisth, as this study is a cross-sectional study, even the verified relationship between second-hand smoking and laryngopathy cannot be considered a causal relationship.

## Conclusion

This population-based epidemiological study verified a significant relationship between second-hand smoking and laryngopathy. More effective anti-smoking policies are required to protect the health of non-smokers as well as smokers. Longitudinal studies are also required in the future to verify the causality between second-hand smoking and laryngopathy.
